# The *N*-myristoylome of *Trypanosoma cruzi*

**DOI:** 10.1038/srep31078

**Published:** 2016-08-05

**Authors:** Adam J. Roberts, Alan H. Fairlamb

**Affiliations:** 1 Division of Biological Chemistry and Drug Discovery, College of Life Sciences, University of Dundee, Dundee, DD1 5EH, UK

## Abstract

Protein *N*-myristoylation is catalysed by *N*-myristoyltransferase (NMT), an essential and druggable target in *Trypanosoma cruzi*, the causative agent of Chagas’ disease. Here we have employed whole cell labelling with azidomyristic acid and click chemistry to identify *N*-myristoylated proteins in different life cycle stages of the parasite. Only minor differences in fluorescent-labelling were observed between the dividing forms (the insect epimastigote and mammalian amastigote stages) and the non-dividing trypomastigote stage. Using a combination of label-free and stable isotope labelling of cells in culture (SILAC) based proteomic strategies in the presence and absence of the NMT inhibitor DDD85646, we identified 56 proteins enriched in at least two out of the three experimental approaches. Of these, 6 were likely to be false positives, with the remaining 50 commencing with amino acids MG at the N-terminus in one or more of the *T. cruzi* genomes. Most of these are proteins of unknown function (32), with the remainder (18) implicated in a diverse range of critical cellular and metabolic functions such as intracellular transport, cell signalling and protein turnover. In summary, we have established that 0.43–0.46% of the proteome is *N*-myristoylated in *T. cruzi* approaching that of other eukaryotic organisms (0.5–1.7%).

The protozoan parasite *Trypanosoma cruzi* is the causative agent of Chagas disease, which continues to be a major health concern in Latin American countries where these parasites are endemic. It is estimated there are ~8 million infected individuals worldwide with over 10,000 deaths annually, mainly due to cardiac disease arising as a consequence of the immune response to the chronic infection^1^,^2^,^3^. Current treatment options are limited to two nitroheterocyclic drugs, benznidazole and nifurtimox, with limited efficacy and toxicity^4^,^5^. Better, safer drugs are needed, but recent clinical trials with ergosterol biosynthesis inhibitors have been disappointing^6^,^7^.

The lifecycle of this parasite consists of three morphologically distinct developmental stages, the epimastigote, trypomastigote and the amastigote (see review^8^). Epimastigotes are the non-infective stage found only in the hind-gut of triatomine bugs. These insects are the primary source of parasite transmission and human infection^9^, although other routes such as blood transfusion, organ transplantation, ingestion of food or drink or vertical transmission are known^8^. Trypomastigotes are the infective and non-dividing stage either found in the faeces of the insect vector or circulating in the bloodstream of a mammalian host. Amastigotes are the replicative forms found intracellularly in a wide range of mammalian cells throughout the body, but can also be detected circulating in the blood^10^,^11^.

The acylation of proteins with fatty acids of various chain lengths occurs in all domains of life (see review^12^). The resulting effect of these modifications can range from regulating biological activity to controlling the subcellular localisation of a protein^13^,^14^,^15^. In particular, myristic acid, which accounts for ~1.5% of the total lipid content of these parasites^16^ can be attached to proteins via a cysteine residue (*S*-myristoylation)^17^ or the *N*-terminal glycine of specific proteins (*N*-myristoylation)^18^,^19^. This latter process is catalysed by the enzyme *N*-myristoyltransferase (NMT; E.C. number 2.3.1.97), which utilizes myristoyl-CoA as an acyl donor and is present in all eukaryotes^19^. Bioinformatic studies have suggested that ~0.5–1.7% of a eukaryotic proteome may be myristoylated^20^,^21^. Although this a conserved biological process, multiple studies have found species-specific differences in the peptide substrates recognised by each NMT homologue, with a greater divergence observed between higher and lower eukaryotes^22^,^23^,^24^. These subtle differences have been exploited to generate species-selective NMT inhibitors for the *Candida albicans*^25^,^26^, *Leishmania spp*^27^,^28^, *Trypanosoma brucei*^29^,^30^ and *Plasmodium falciparum* homologs^28^.

Previous work from our lab has demonstrated that NMT is both a druggable and essential target in *T. cruzi*^31^, similar to the related parasites *T. brucei*^30^ and *L. major*^27^,^32^. The specific inhibition of *N*-myristoylation in *T. cruzi* using the potent NMT inhibitor DDD85646^30^, consequentially led to a reduction in parasite proliferation in the epimastigote stage^31^. In order to ascertain if *N*-myristoylated proteins themselves may be potential therapeutic targets in *T. cruzi*, the *N*-myristoylome first needs to be identified and characterised. This study has sought to overcome the uncertainty associated with identifying *N*-myristoylation by bioinformatics alone using a bead-based chemical proteomic strategy. This approach has led to the robust identification of 50 proteins involved in a variety of cellular pathways and functions.

## Results

### *T. cruzi N*-myristoylation

**Figure 1:**
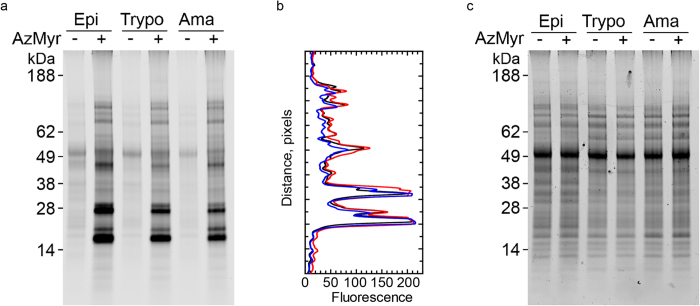
Comparative *N*-myristoylation throughout the *T. cruzi* life cycle. (**a**) The incorporation of azidomyristate (AzMyr) into the epimastigote (Epi), trypomastigote (Trypo) and amastigote (Ama) proteomes was visualised using click chemistry. (**b**) Fluorescence intensity profile of the epimastigote (red), trypomastigote (blue), amastigote (black) *N*-myristoylated proteins, assessed by in-gel fluorescence. (**c**) Coomassie blue stained gel.

Previous work from our lab has identified that NMT is continuously expressed throughout the lifecycle of the parasite^31^. To determine the relative expression of *N*-myristoylated proteins in all life-cycle stages, the incorporation of the myristic acid analogue, azidomyristate, was monitored using click chemistry and SDS-PAGE as a measure of *N*-myristoylation. In all samples labelled with or without azidomyristate, the fluorescent dye was found to cross-react with a non-specific band at ~50 kDa. Azidomyristate was incorporated into at least 10 bands visible in SDS-PAGE gels in epimastigotes, trypomastigotes and amastigotes (Fig. 1a). These bands were insensitive to base treatment suggesting that they had been incorporated via an N-terminal amide bond, rather than by *S*-myristoylation^17^. Analysis by densitometry revealed at least 18 bands common to all stages, some of which varied in intensity between the lifecycle stages (Fig. 1b) despite an equal amount of protein loaded as assessed by Coomassie blue staining (Fig. 1c). Thus, our results demonstrate that there are only minor stage-specific variations in the labelling of the most abundant *N*-myristoylated proteins.

### *N*-myristoylation is co- rather than post-translational

**Figure 2:**
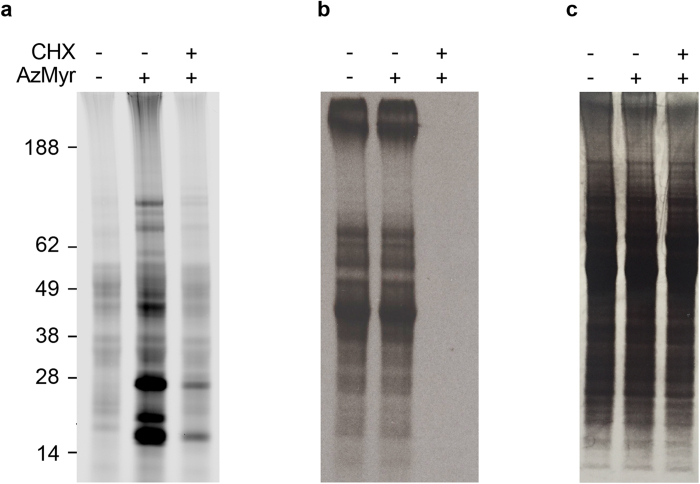
Co- versus post-translational *N*-myristoylation. Epimastigotes were pre-incubated in the presence and absence of cycloheximide (CHX) prior to labelling with azidomyristate (AzMyr) and subsequent visualisation by in-gel fluorescence (**a**). L-[^35^S]-methionine incorporation was measured by autoradiography (**b**). Coomassie blue stained gel showing equal loading (**c**).

This modification has been reported to occur post-translationally during apoptosis in mammalian cells, where proteolytic cleavage reveals a hidden myristoylation motif 33,34. To elucidate if this can occur post-translationally in *T. cruzi*, incorporation of azidomyristate was assessed in epimastigotes where nascent protein synthesis was inhibited by pre-treatment with cycloheximide. Examination by microscopy and Live/Dead assay revealed cycloheximide treatment had no effect on the viability of the cells. *N*-myristoylation in the epimastigote was greatly reduced or abolished to below the limits of detection in the presence of cycloheximide for the majority of bands (Fig. 2a). However, two out of the three most prominent azidomyristoylated bands at 24 and 16 kDa were partially resistant to cycloheximide treatment in epimastigotes. To assess if this was a failure to inhibit nascent protein synthesis, concurrent labelling with *L*-[^35^S]-methionine was undertaken (Fig. 2b). This revealed no detectable protein synthesis in parasites that had been treated with cycloheximide possibly suggesting that these bands may be the result of post-translational myristoylation.

### The identification of *N*-azidomyristoylated proteins

**Figure 3:**
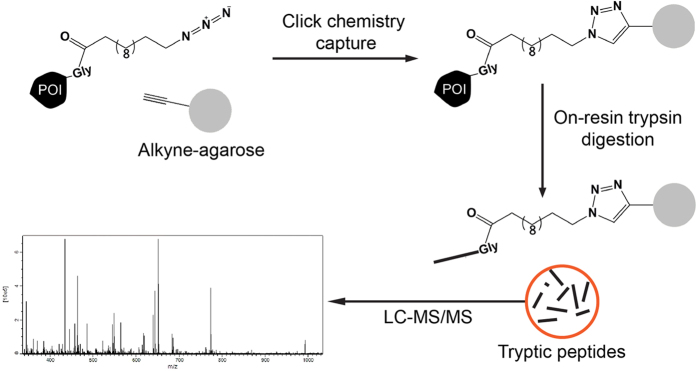
Enrichment strategy for the identification of the *T. cruzi N*-myristoylome (**a**) *N*-azidomyristoylated proteins were directly captured onto an alkyne-agarose resin using click chemistry. Stringent washing of the resin under denaturing conditions is designed to remove non-specific contaminants. Enriched proteins were digested on resin with trypsin and the recovered peptides were analysed by LC-MS/MS.

**Figure 4:**
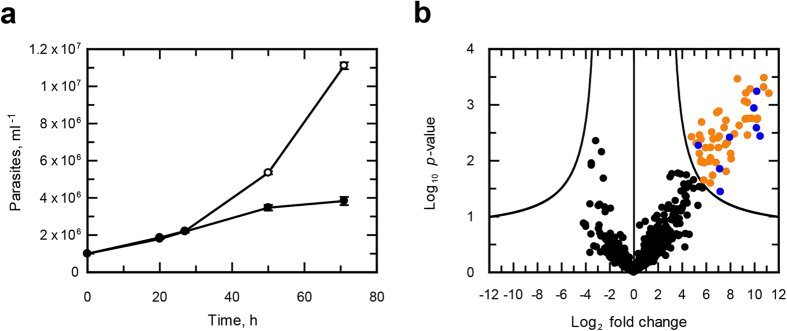
Proteomic analysis of the *T. cruzi N-*myristoylome. (**a**) Growth of *T. cruzi* epimastigotes in RTH/FCS supplemented with DMSO (open circles) or azidomyristate (closed circles). (**b**) Volcano plot displaying the enrichment of *N*-myristoylated proteins from three independent biological replicates. The line shows the significance threshold for the t-test with permutation-based FDR (*q* < 0.05), with significantly enriched proteins absent from all controls (orange), significantly enriched proteins identified in at least one control (blue) and non-significant (black).

To identify the proteins undergoing this modification, we directly captured and enriched *N*-azidomyristoylated proteins using bead-based click-chemistry and on-resin trypsin digestion followed by analysis by mass spectrometry (Fig. 3). Epimastigotes labelled with azidomyristate grew at the same rate as the control for the first 27 h, after which the presence of this analogue was detrimental to parasite growth (Fig. 4a). Epimastigotes have a doubling time of approximately18–22 h, so a labelling period of 20 h was selected for label-free quantitation as this would maximise labelling of cell cycle-dependent proteins and proteins with a slow turnover, whilst avoiding cell toxicity. Enrichments were made from urea-solubilised whole cell lysates of control or azidomyristate-labelled parasites carried out from three independent biological replicates. Stringent washing and hydroxylamine treatment of the resin was carried out to remove *S*-myristoylated proteins, non-specific binding proteins and missing label-free quantitation (LFQ) values, imputed from a normal distribution. Significance was assessed using a permutation based t-test, identifying 56 proteins to be significantly (*t*-test, 250 permutations, S0 = 2, FDR *q* < 0.05) enriched over the controls (Fig. 4b orange and blue). Removal of the imputed values showed that the majority of enriched proteins had not been identified in any control experiment (Fig. 4b, orange), with 8 out of 56 having been identified in at least one control (Fig. 4b, blue). Of the 56 enriched proteins identified 79% were predicted to have an *N*-terminal methionine followed by a glycine residue (Table S2). Overall, this represents a 15-fold enrichment over the database average of 5.26% of proteins commencing with MG, as would be expected for the enrichment of *N*-myristoylated proteins from this parasite.

**Figure 5:**
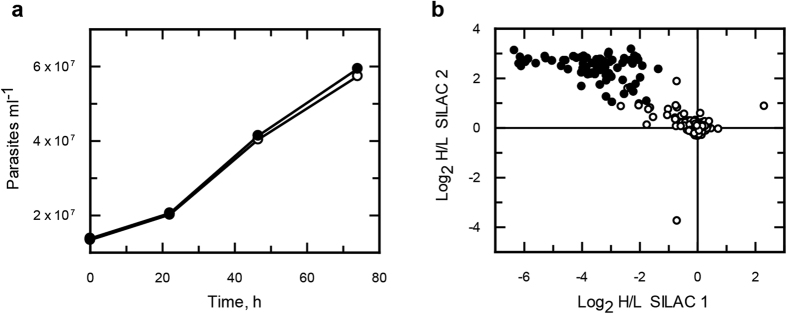
SILAC studies of *N*-azidomyristoylation in *T. cruzi* epimastigotes. (**a**) Epimastigotes were grown in SDM-79 medium supplemented with light (open circles) or heavy (closed circles) isotopically labelled amino acids. (**b**) Log2 H/L ratios plotted for SILAC experiments 1 (x axis) and 2 (y axis).

To minimise sample handling and processing errors, we opted to perform a further 2 experiments using stable isotope labelling of cells in culture (SILAC) to determine if there were *N*-azidomyristoylated proteins that also bound non-specifically to beads. As the culture medium RTH/FCS is undefined (contains trypticase peptone), the more chemically defined medium SDM-79 was used for isotopic labelling as it has been reported to support the growth of epimastigotes^35^ and has been successfully used for SILAC studies in *T. brucei*^36^. The parasites were found to grow at similar rates in both the heavy and light media (Fig. 5a). Enrichments were made from the isotopically-labelled parasites with the labels swapped for the second enrichment. The data was adjusted to the mode value for both enrichments (Fig. S1). Significance A and B tests only identified a single protein with statistical significance, possibly due to the non-normal distribution of log_2_ H/L ratios. Under the assumption that the majority of protein ratios would remain unchanged, arbitrary cut-off values for enrichment of < 1 and > 1 for experiments 1 and 2 respectively were assigned (Fig. S1). Overall, 77 proteins were identified in both SILAC biological replicates (Fig. 5b and Table S2), 58% of which were annotated to have a glycine at position 2.

As a result of the direct capture-enrichment approach, the *N*-terminal azidomyristoylated-peptide is retained on the agarose, so a secondary digestion of both SILAC samples with polymyxin acylase was carried out with the aim of releasing these peptides^37^. Although peptides were recovered from replicates 1 and 2 (2 and 4 peptides, respectively, see Table S3), these did not possess an *N*-terminal glycine residue. Only one of these peptides matched a protein enriched in one of the replicates, (K4EBF6, 846 amino acids). A BLAST search of this protein against the CL-Brener genome identified a hypothetical gene (TcCLB.509213.40) encoding a protein of 748 residues commencing with MG. This contained a motif (pfam08432: Vfa1), postulated to be involved in regulating the trafficking of other proteins to the endocytic vacuole. However, alignment of the orthologues from Silvio and Dm28c with CL-Brener strains revealed markedly different N-terminal sequences with MG only in the latter strain, despite near identity over the 720 amino acids towards the C-terminus. Similar BLAST searches with the other 5 peptides did not reveal any genes encoding proteins with an upstream MG start site. Most likely, their identification is probably a result of carryover from the initial experiments, despite extensive washing of the resin. This suggests that polymyxin acylase is not capable of de-acylating, click-immobilised acyl peptides, possibly due to steric hindrance. Further experiments are required to substantiate this hypothesis.

### Analysis of enriched proteins

**Figure 6:**
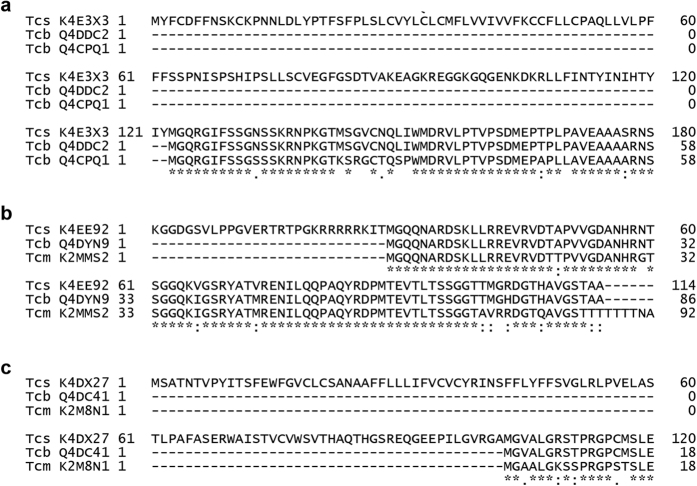
Alignment of non-MG proteins with *T. cruzi* homologs. Alignments of proteins enriched in SILAC and LFQ experiments that appear to have been incorrectly annotated. Proteins are aligned against sequences of other strains available in UniProt.

By combining both the label-free and SILAC data, we found 48 consistently enriched proteins of which >80% were annotated to have an *N*-terminal MG (Table S2). Those proteins with a predicted function or suspected misannotation are listed in Table 1. Some of these have previously been reported to be *N*-myristoylated in other organisms, including CAP5.5 that has been previously identified in *T. cruzi*^31^. However, two proteins that have been reported to be *N*-myristoylated in *T. cruzi* were not enriched in all experiments. The flagellar calcium binding protein (FCaBP)^38^ was significantly enriched (*t*-test, 250 permutations, S0 = 2, FDR *q* < 0.001) in the label free studies and identified, but not quantified in the SILAC experiments. In the case of the phosphoinositide phospholipase C^39^, this was identified in only 2 out of 5 experiments. One of the consistently enriched sequences was annotated without an *N*-terminal methionine in the X10/1 genome, therefore proteins without an annotated N-terminal MG were searched using BLAST to determine the likelihood of correct annotation. This revealed that 3 out of the 9 non-MG proteins may have been incorrectly curated in the X10/1 proteome, because sequences from other *T. cruzi* genome strains had alternative initiation of translation sites commencing with MG downstream of the X10/1 sequences (Fig. 6a–c). This observation is supported by the fact that no peptides were identified upstream of the potential downstream MG sites for any of these potentially misannotated proteins.

### Profiling the enrichment of myristoylated proteins in the presence of an NMT inhibitor

**Figure 7:**
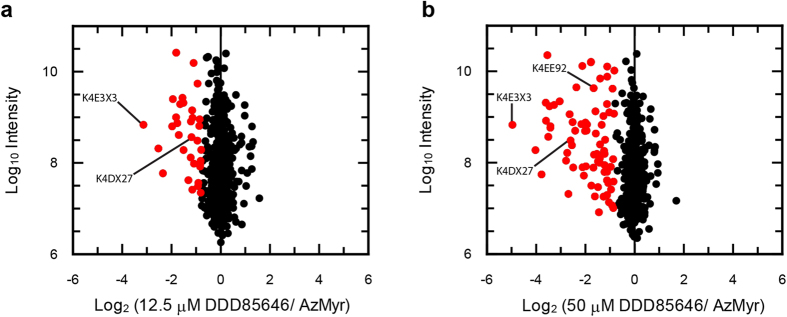
Enrichment of *N*-myristoylated proteins from DDD856464 treated parasites. Enrichment of azidomyristate labelled proteins from epimastigotes also treated with 12.5 μM (**a**) and 50 μM (**b**) of the NMT inhibitor DDD85646. Significantly enriched proteins are shown in red as assessed using significance (**b**) (FDR *q* < 0.05).

The NMT inhibitor DDD85646 has previously been shown to inhibit *N*-myristoylation in *T. cruzi* and other parasites by both gel-based and label-free proteomic approaches^31^,^40^,^41^. Thus, we attempted to confirm the incorrect-annotation of the enriched proteins lacking an apparent *N*-terminal MG by incubating light, medium and heavy labelled parasites with azidomyristate in the absence and presence of DDD85646 (0–50 μM). Co-incubation of DDD85646 at ~2 or 8 × EC_50_, led to the significant (Significance B, left tailed Benjamini-Hochberg FDR, *q* < 0.05) under-enrichment of 33 and 75 proteins, respectively, in comparison with the no drug control (Fig. 7a,b and Table S4). Proteins most sensitive to the inhibition of NMT included an ADP-ribosylation factor and homologs of small myristoylated proteins as well as multiple uncharacterised proteins. Comparison between the two drug concentrations also revealed a dose-dependent effect of DDD85646 on the enrichment for 57 proteins supporting our previous findings^31^. At both concentrations of inhibitor, the majority of differentially enriched proteins were annotated with an N-terminal MG (82–84%), inferring their myristoylation status (Table S4). Interestingly, the three proteins predicted to be incorrectly annotated (Fig. 6a–c) were significantly less enriched in the presence of the higher concentration of inhibitor, with two out of the three under-enriched at the lower concentration also. These data add further evidence that they have been incorrectly curated within the *T. cruzi* genome and are in fact bona-fide *N*-myristoylated proteins.

Overall, the use of an NMT inhibitor combined with SILAC identifies a higher percentage of substrates with greater confidence than that by SILAC based enrichment alone. This is evident by the number of proteins annotated with an N-terminal glycine and that DDD85646 inhibits the *N*-myristoylation of CAP5.5, a known substrate for *T. cruzi* NMT^31^.

### Theoretical versus actual enrichment

Proteins enriched in at least two experimental designs were deemed to be consistently enriched with a total of 56 identified after the removal of the duplicate K4E681 (Fig. S2). Thirty two of these were detected in the three orthogonal experimental approaches. Fifty of these were found to have an N-terminal glycine or predicted to have one due to poor curation (Table S5). These consistently enriched proteins were compared to their bioinformatics predictions using Myristoylator and both settings of the NMT Myr Predictor. However, these programmes were only able to unanimously predict the *N*-myristoylation status for about half of the proteins with an experimentally determined or predicted N-terminal glycine (Table S6). Individually, the results were marginally better with Myristoylator, predicting 31 out of 50 proteins with a high confidence compared to 26/50 and 21/50 using the all eukaryotes and fungi only settings of the Myr Predictor. Comparison of the theoretical masses of the enriched proteins matched the distribution of bands by in-gel fluorescence, with the largest number of proteins identified having a mass less than 40 kDa. A large proportion of these enriched proteins have no known function; however from the limited Gene Ontology annotation, it is clear that these proteins are involved in a variety of cellular pathways and functions.

As there have recently been reports of the enrichment of *N*-myristoylated proteins from the malaria parasite *P. falciparum*^41^ and *L. donovani*^40^, we attempted to identify orthologues in the published data from the related parasites. As one would expect, we identified more homologs in *Leishmania* than malaria, although 8 potential homologs were identified in both (Table S7). These included two ADP-ribosylation-factor (ARF) family members, a proteasome regulatory subunit and several protein phosphatases.

## Discussion

With the use of a chemically tractable, metabolic label, we have identified 56 putative *N*-myristoylated proteins in *T. cruzi*, of which 32 were detected in the three orthogonal experimental approaches. Forty six of the 56 begin with the amino acids MG, with an additional 4 predicted to be MG by alignment with other *T. cruzi* homologues. The enrichment of the latter 4 proteins was reduced by chemical inhibition of NMT by the potent and specific inhibitor, DDD85646. Of the remaining 6 proteins, 4 have strong putative mitochondrial targeting signals (MitoProt scores 0.86–0.99)with predicted cleavage sites, but none of the amino acids following the cleavage sites are glycine. Thus, we can robustly assign 50 proteins as being *N*-myristoylated in *T. cruzi,* extending our previous report showing that protein myristoylation can be reduced or abolished in these parasites in a dose-dependent manner by the potent and specific NMT inhibitor DDD85646^31^. Studies on *T. cruzi* prior to this investigation have been limited to validating one protein at a time, or to making assignments of myristoylation using current bioinformatic prediction software^21^,^42^. Despite the limitations of the current prediction programs, our study identified 10 homologues (Table S2) previously predicted to be enriched in membrane fractions^43^. The enrichment of FCaBP, identified as myristoylated in the parasite^38^, and of CAP5.5, shown to be a substrate for *T. cruzi* NMT^31^, demonstrates the ability to identify *N*-myristoylated proteins from this organism.

Notably, similar enrichments have recently been reported for mammalian cells^44^, *Plasmodium falciparum*^41^ and the related parasite *Leishmania donovani*^40^. In the case of the latter, these authors noted that, although both azido and alkynyl myristic acid analogues were incorporated, the alkynyl was superior due to its lower background labelling and more favourable incorporation^40^. A comparison was not carried out in *T. cruzi* due to the limitations of the commercial kit used in this study. Nonetheless, we were able to identify the homologs of several proteins from the *Leishmania N*-myristoylome (Table S7). Similarly, we also identified several proteins from an enrichment carried out from *Plasmodium* parasites^41^ (Table S7). A very recent study on the *N*-myristoylome of *T. brucei*^45^ also shows enrichment of about half of the proteins identified here (Table S7) underlining common, as well as distinct, elements in the biology of these related parasites.

The downstream analysis of the 56 enriched proteins was complicated by poor annotation and poor curation of the proteome. As an example, the three ADP-ribosylation factors (ARF) and ARF-like proteins were annotated with conflicting annotations when compared to the *Leishmania* ARF proteins (Table S7). Another example is the identification of the uncharacterised fragment K4EE92 appears to have a Pfam domain consistent with the β-subunit of AMP-activated protein kinase (AMPKβ), a known myristoylated protein in eukaryotes that is involved metabolic stress response and regulation^13^. *T. brucei* AMPKβ also possesses a putative *N*-myristoylation signal and is exclusively associated with glycosomes and the flagellum^46^. The three protein phosphatase 2C family members (or metal-dependent protein phosphatases, PPM in more recent terminology) are most similar to PPM1A in the mouse genome. Interestingly, these protein phosphatases have been shown to require *N*-myristoylation in order to dephosphorylate the α-subunit of AMPK^47^, but it is not known if a similar interaction occurs in *T. cruzi*.

*N*-myristoylation involves covalent attachment to the N-terminal glycine. The glycine can either be revealed during protein synthesis by removal of the initiator methionine residue by methionine amino peptidases (co-translational), or during apoptosis where proteolysis by caspases reveals hidden *N*-myristoylation motifs (post-translational)^34^,^44^. Although the vast majority of myristoylation appears to be co-translational in our experiments, two of the three most prominent bands appear to be post-translational. However, strong evidence for apoptosis or necrosis in these parasites is lacking^48^ and incidental cell death can be eliminated as a possible cause since we found no evidence of loss of cell viability in our experiments. *N*-myristoylation is generally regarded as irreversible, due to the absence of enzymes capable of specifically removing the myristoyl moiety from the N-terminal glycine residue. However, there are exceptions. For example, *N*-myristoylation of the protein kinase C MARKS in brain synaptosomes has been reported to occur in the absence of protein synthesis^49^. Moreover, NMT can catalyse the de-myristoylation of a peptide substrate^50^. This reverse reaction occurs at a rate 1,500 to 3,000-fold lower^50^ than *N*-myristoylation of the peptide. Thus, the failure to completely abolish *N*-myristoylation by complete inhibition of *de novo* protein synthesis could be explained by the de-acylation of myristic acid and re-acylation with azidomyristate-CoA.

In line with the current knowledge in other eukaryotes, it appears that protein myristoylation in *T. cruzi* is involved in a variety of cellular pathways and functions, including: membrane trafficking and cellular transport (ARFs and ARLs); cell signalling and regulation of metabolic processes (AMPK and PP2Cs); and cytoskeletal remodelling and protein turnover (calpains and the proteasome). However, the majority of proteins are currently uncharacterised and have no known functions aside from the identification of a few Pfam domain predictions. It therefore appears that there is a large proportion of *N*-myristoylation biology that has yet to be investigated and understood, which may in future provide novel insights into the biology of this parasite.

In summary, this is the most comprehensive enrichment and identification of *N*-myristoylated proteins from this parasite reported to date, as far as we are aware. The genome of the CL-Brener strain is a hybrid of Esmeraldo-like and non-Esmeraldo-like haplotypes comprising 23,216 gene models encoding 21,170 proteins, of which over 23% of the annotated genes in the genome are members of large gene families, such as trans-sialidases and mucins^51^. Based on the CL-Brener genes listed in Table S5 as probably commencing with MG, we estimate 0.43% of the proteome is *N*-myristoylated. A similar value of 0.46% is obtained from the Silvio X10/1 predicted proteome (10,876 proteins). These values are marginally lower than the 0.5–1.7% predicted in other eukaryotic proteomes^20^,^52^,^53^. This may be a result of only capturing the most abundant *N*-myristoylated proteins in the parasite, which, given the failure to identify two known *N*-myristoylated proteins, suggests that there may be more novel proteins yet to be identified.

## Methods

### Cell culture

Epimastigotes of the *T. cruzi* strain Silvio X10/7 clone A (MHOM/BR/78/Silvio; clone X10/7A) were grown in RTH medium supplemented with 10% foetal calf serum (FCS, PAA) at 28 °C^54^. Late-stage cultures containing a mixture of epimastigotes and trypomastigotes were used to infect Vero cell monolayers overnight with a ratio of 10 parasites per Vero cell. Non-invaded parasites were washed off and after 5–6 days a relatively pure population of trypomastigotes was obtained by removing the supernatant. Amastigotes were purified as previously described^55^. Vero cells were cultured in Dulbecco’s Modified Eagle Medium supplemented with 10% FBS (DMEM/FBS) as described elsewhere^56^.

### Stable Isotope Labelling with Amino Acids in Cell Culture (SILAC)

SDM-79 medium depleted of both arginine and lysine was reconstituted at the original concentration with either the naturally abundant (R_0_K_0_), medium labelled (R_6_K_4_) or heavy labelled (R_10_K_8_) amino acid isotopes (L-arginine.HCl and L-lysine.2HCl, CK Gas Products). Dialysed FCS (10% v/v) was used in place of standard FCS and the medium supplemented with 100 μM putrescence before epimastigotes were adapted for growth in the heavy or light media as reported for the original SDM-79^35^.

### Visualising *N*-azidomyristoylated proteins

Parasites were labelled with 50 μM 12-azidododecanoic acid (azidomyristate) for 6 h in either RTH/FCS or DMEM/FCS. Extracts from 5 × 10^7^ cells were made in trypanosome lysis buffer (50 mM Tris-HCl, pH 7.4,150 mM NaCl, 1% sodium deoxycholate, 0.1% SDS, 1% Triton X-100 and a cOmplete mini EDTA-free protease inhibitor cocktail tablet) by incubating on ice for 1 h followed by biological inactivation by freeze thawing three times. *N*-azidomyristoylated proteins from lysates were labelled with IRDye 800CW alkyne, using the Click-iT^®^ protein reaction buffer kit (Molecular probes)^31^. Lysates were separated by SDS-PAGE on a 4–12% NUPAGE in MES SDS buffer (Invitrogen), and treated with 1 M NaOH for 1 h to remove S- and O- myristoylation^17^. Gels were imaged by in-gel fluorescence with an Odyssey SA infrared imager (LI-COR Biosciences). For the inhibition of protein synthesis, parasites were pre-incubated with 50 μg ml^−1^ cycloheximide for 30 min prior to labelling with azidomyristate. Fluorescence intensity profiles were extracted using ImageJ (http://imagej.nih.gov/ij/) and plotted as a factor of distance migrated through the gel.

### *L*-[35S]-methionine labelling

Parasites were washed three times in phosphate buffered saline (PBS 137 mM NaCl, 2.7 mM KCl, 10 mM Na_2_HPO_4_ and 1.8 mM KH_2_PO_4_) and resuspended in methionine-free RTH/FCS supplemented with or without 50 μg ml^−1^ cycloheximide for 30 min prior to the addition of 10 μCi ml^−1^ L-[^35^S]-methionine (Perkin Elmer). Lysates were made in Laemmli buffer and separated by SDS-PAGE as described above. The gel was treated with En3Hance solution (Perkin Elmer) as per manufacturer’s protocol, dried and exposed to BioMax MS film (Kodak) using a TransScreen LE (Kodak).

### Live/dead assay

Parasites were washed in PBS and incubated with the live/dead staining mix (5μg ml^−1^ Hoechst 33342 and 2 μl ml^−1^ Red Dead Molecular probes) for 15 min at room temperature. Parasites were harvested and resuspended in PBS supplemented with 1% formaldehyde. Cells were imaged with an Axiovert 200 inverted microscope using the appropriate filters, analysing 10 fields of view.

### Enrichment of *N*-azidomyristoylated proteins

Parasites were seeded at a density of 5 × 10^6^ cells ml^−1^ and grown in the presence of 50 μM azidomyristate or DMSO for 20 h (label free) or 12 h (SILAC). Cells were harvested by centrifugation (15 min, 1620 × g and 4 °C) and washed twice in PBS to remove the excess label (SILAC parasites were mixed in a 1:1 ratio prior to the first wash). *N*-Azidomyristoylated proteins were enriched using the protein enrichment kit (Molecular probes, Life Tech). Parasites (2 × 10^9^) were resuspended in 850 μl of the supplied urea lysis buffer and parasites inactivated by three cycles of freezing and thawing, prior to sonication. Soluble protein was obtained after centrifugation (5 min, 10,000 g at 4 °C), and 800 μl was used for the click chemistry immobilisation as per the manufacturers recommendations. Non-specifically interacting proteins were removed by washing the resin with the supplied SDS wash buffer followed by equilibration with H_2_O. The agarose was incubated with neutral hydroxylamine to remove *S*-azidomyristoylated proteins^17^, and then bound proteins were reductively alkylated and further processed according to the suggested protocol.

### Mass spectrometry

Agarose immobilised proteins were digested on-resin with trypsin. Protein digestion and the subsequent sample processing for analysis by mass spectrometry was carried out by the FingerPrints proteomics service (http://proteomics.lifesci.dundee.ac.uk, University of Dundee). Mass spectra were acquired on an LTQ Orbitrap Velos Pro as described in the supplementary methods. Thermo Xcalibur raw files were processed with MaxQuant^57^ version 1.5.0.0 which incorporates the Andromeda search engine^58^. Protein identifications were made from a custom *T. cruzi* database comprising the “complete” proteome retrieved from UniProt which contains predicted sequences from the CL Brener and Silvio X10/1 strains and *T. cruzi marinkellei* (30,048 entries) in addition to a database of common laboratory contaminants. Searches were carried out using a MS tolerance of 4.5 ppm, an MS/MS tolerance of 0.5 Da and allowing for up to two missed trypsin cleavages. Carbamidomethylation of cysteine residues was set as a fixed modification, with the oxidation of methionine and *N*-acetylation were set as variable modifications. Assigned peptides were required to be a minimum of 7 amino acid residues in length. Only unique peptides were used to calculate LFQ and SILAC ratios. False discovery rates (FDR) were calculated to be < 0.01 by searching a decoy database consisting of reversed sequences. The raw and processed mass spectrometry data have been deposited with the ProteomeXchange Consortium (http://www.proteomexchange.org/)^59^ using the PRIDE uploader^60^ under the identifier (PXD002970). Additionally, peptide identifications and protein groups have been included in Table S1. Subsequent data processing and analysis was performed using Perseus v.1.5.16.

### LFQ analysis

Label-free data were processed with the match between runs feature enabled in MaxQuant using the settings described above. Data were filtered to remove proteins only identified by site, matching reverse sequences and contaminant proteins, prior to transformation to log_2_ LFQ intensities. The data were filtered to require a minimum of 3 valid values prior to the imputation of missing values using a normal distribution (0.1 width, 1.8 downshift). Statistical significance was assessed using a modified *t*-test (250 permutations, FDR = 0.05, S0 = 2)^40^.

### SILAC enrichment analysis

The data sets were filtered to remove proteins only expressed in one experiment. Raw SILAC H/L ratios were log2-transformed prior to normalisation to the mode ratio in Perseus. Using histograms of the label swap experiments, an arbitrary enrichment cut-off of >1 log_2_-fold change was assigned for each of the normalised datasets.

### DDD85646 SILAC enrichment experiment

WT parasites labelled with R_0_K_0_, R_6_K_4_ or R_10_K_8_ were incubated with 50 μM azidomyristate for 12 h also in the presence of 0, 12.5 and 50 μM DDD85646^30^ respectively. An equal number of heavy, medium and light labelled parasites were combined and processed as described above for the enrichment of *N*-azidomyristoylated proteins. The dataset is the result of two technical replicates, processed in Max Quant using the appropriate labels using both unique and razor peptides for quantification. Using the normalized data, significant (FDR q < 0.05) differences in the enrichment of proteins from the experimental conditions were assessed in Perseus, using a one tailed Significance B test with a Benjamini-Hochberg FDR truncation implementing a 0.05 threshold value.

### Polymyxin acylase digestion

After digestion with trypsin, the agarose was extensively washed with 20% acetonitrile to remove remaining non-covalently bound peptides and then equilibrated in 50 mM phosphate buffer (pH 8.0). Following this, a secondary digestion with 6.4 U of polymyxin acylase (Wako Chemicals) was carried out at 37 °C for 24 h^37^. Peptides were extracted by the FingerPrints proteomics service, University of Dundee, desalted and analysed by LC-MS/MS. Data were analysed using a custom programmed enzyme with the specificity of trypsin/P with the addition of an enzyme that cleaved between the C-terminus of methionine and the N-terminus of glycine.

### Bioinformatic searches

Prediction of *N*-myristoylation was assessed using Myristoylator^42^ and the NMT Myr Predictor^21^. Comparison of enriched proteins was carried by creating custom BLAST databases in CLC workbench using the published results from *L. donovani Plasmodium falciparum* and *T. brucei.* BLAST searches were carried out using an E-value cut-off of 1 using Blosum62 with a gap cost of existence 11, extension 1.

## Additional Information

**How to cite this article**: Roberts, A. J. and Fairlamb, A. H. The *N*-myristoylome of *Trypanosoma cruzi*. *Sci. Rep.* **6**, 31078; doi: 10.1038/srep31078 (2016).

## Supplementary Material

Supplementary Information

Supplementary Table S1

Supplementary Table S2

## Figures and Tables

**Table 1:** Selected proteins enriched in at least 2 experiments with predicted function or suspected misannotation.

UniProt Accession	Protein names	First 2 aa	Possible incorrect annotation in *T. cruzi* genomes	Number of genes in CL-Brener	Reported *N*-myristoylation in other organisms
Q4D7Y8	ADP-ribosylation factor 1 (ARF1)	MG		4	*T. brucei*^61^
Q4DPJ1	ADP-ribosylation factor (ARL1)	MG		2	*T. brucei*^62^
Q4DZM9	ADP-ribosylation factor-like protein	MG		1	
Q4CV42	Calpain-like cysteine peptidase	MG		1	*L. major*^63^
Q4CW64	Calpain-like cysteine peptidase	MG		1	*L. major*^63^
K4E5Y1	Cytoskeleton associated protein CAP5.5	MG		3	*T. brucei*^64^
K4DT87	Dynein heavy chain, putative	MG		1	
K4E5P0	Fatty acyl CoA synthetase 2	MG		2	
K4E8Y0	I/6 autoantigen (microtubule cytoskeleton)	MP	No, all strains	2	
K4E595	Nitrate reductase	MG		1	
K4EE92	5′-AMP-activated protein kinase subunit beta, putative	KG	Yes, Silvio	2	*M. musculus*^13^
K4E583	Protein phosphatase 2C	MG		1	
K4DTB6	Protein phosphatase 2C	MG	Yes, CL-Brener	1	
Q4E4N2	Protein phosphatase 2C	MG		1	
Q4D0B9	Proteasome regulatory ATPase subunit 2	MG		2	*S. cerevisiae*^14^ *T. brucei*^45^
Q4CWV8	Iron-sulphur assembly protein 2, putative	ML	No, all strains	2	
K4DXT8	Zinc finger protein	MG		2	
K4DX27	Uncharacterized protein	MS	Yes, Silvio	2	
K4E0J9	Uncharacterized protein	MR	ML 1/2 orthologues CL-Brener	2	
K4E0P3	Uncharacterized protein	MM	No, all strains	2	
K4E3X3	Uncharacterized protein	MY	Yes, Silvio	2	

Predicted misannotation was determined by BLAST searching against other *T. cruzi* homologues (see Fig. 6 for alignments). The complete list of 42 proteins with links to orthologues in the CL-Brener genome is provided in Table S5.
