# Supplementary material for: The N-myristoylome of Trypanosoma cruzi
Source: Sci Rep. 2016 Aug 5;6:31078. doi: 10.1038/srep31078 (PMC4974623; doi:10.1038/srep31078)
Supplement: Supplementary Information [file srep31078-s1.pdf]

Supplementary Information

The *N*-myristoylome of *Trypanosoma cruzi*

Adam J. Roberts and Alan H. Fairlamb *

Division of Biological Chemistry and Drug Discovery, College of Life Sciences, University of Dundee, Dundee, DD1 5EH, UK

* **Corresponding author:** Email a.h.fairlamb@dundee.ac.uk. Tel: 01382 385 155 Fax: 01382 385 542

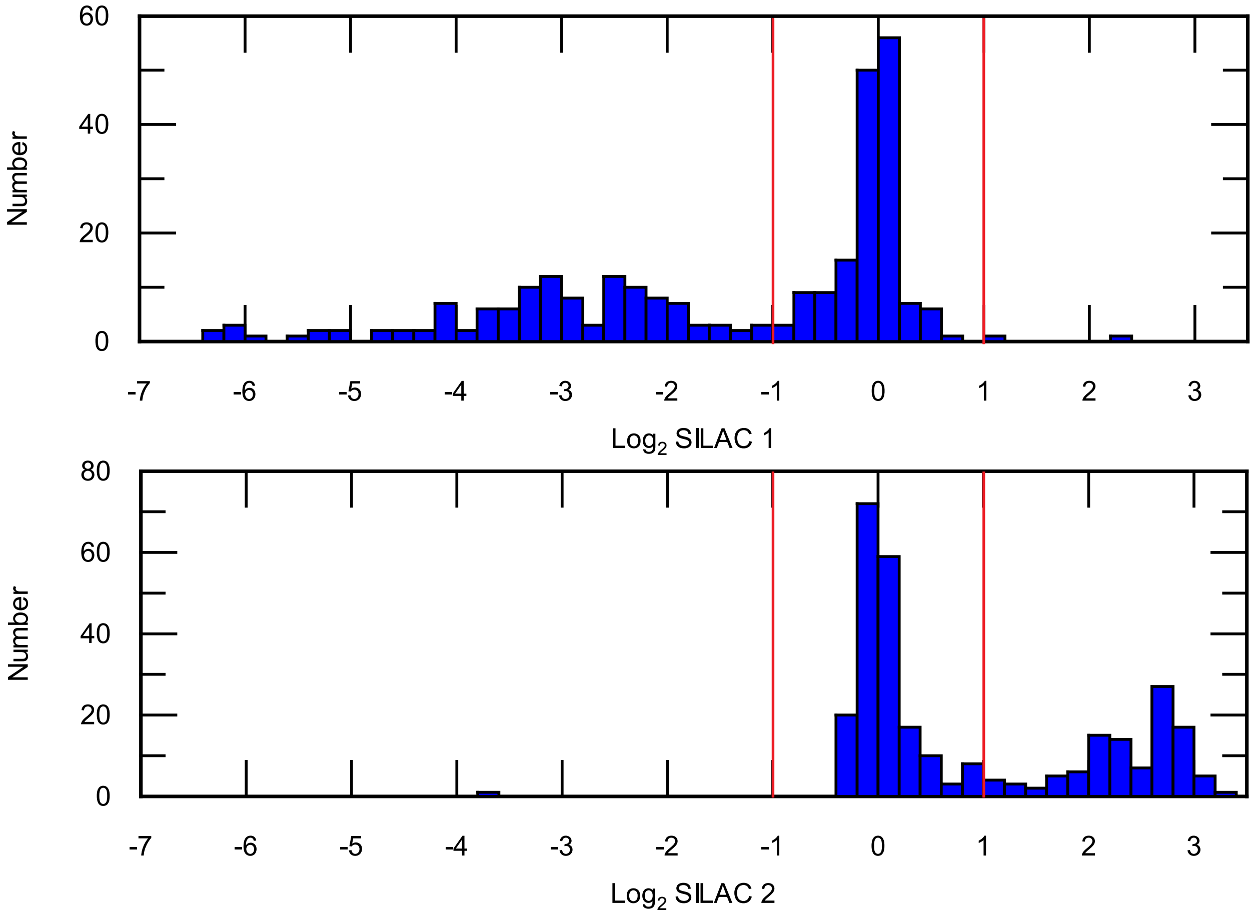

Figure S1 Histogram plots of the Log_2 transformed SILAC ratios for experiments 1 and 2. AzMyr was used to label the isotopically light parasites in experiment 1 and the heavy parasites in experiment 2. Log2 H/L ratios were normalised to the mode value and plotted on the x axis, with the frequency on the y axis. Arbitraty cut-off boundaries for enrichment are marked by the red lines.

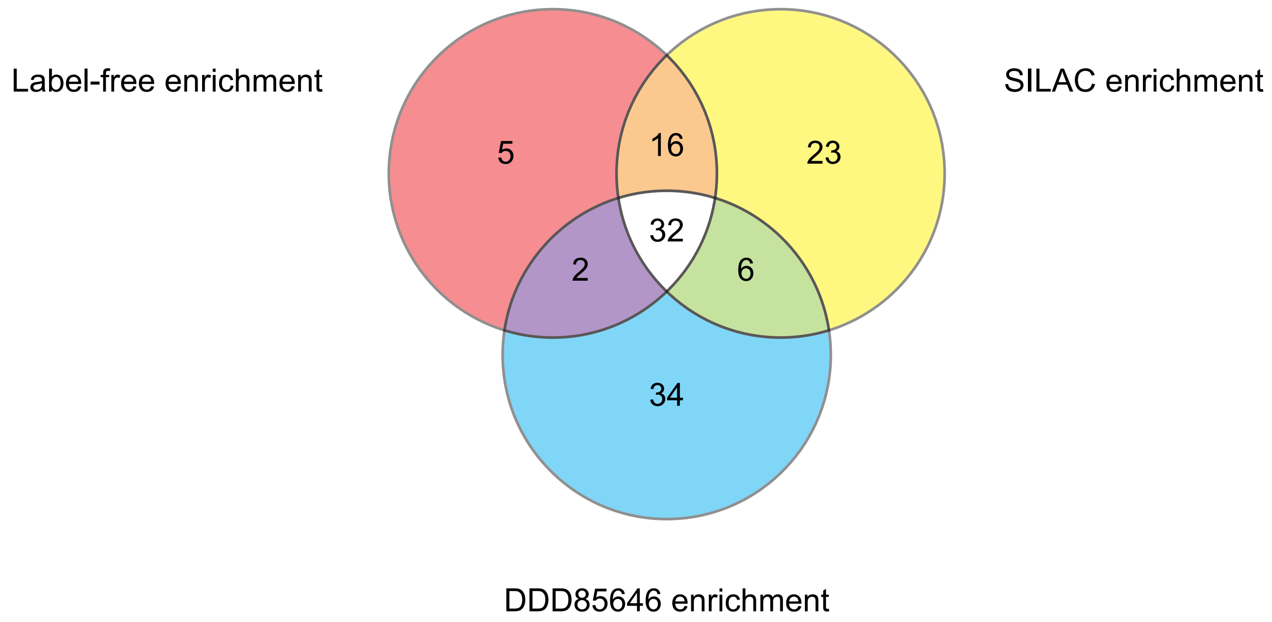

Figure S2 Venn diagram showing the enrichment of proteins from the three experimental approaches.
A total of 32 proteins were ifentified in all three experiments, with all annotated or predicted to have an N-terminal MG. 56 proteins were identified in atleast 2 experiments with the majority predicted to have the N-termianl MG, required for *N*-myristoylatiom.

Table S1 List of protein groups and peptides identified in label-free, SILAC enrichment and DDD85646 experiments. Data is provided as an .xls file.

Table S2 List of proteins enriched in label-free experiments (LFQ) and or SILAC experiments. Data is provided as an .xls file.

UniProt	Score	Sequence	Experiment	Annotated Ms/Ms spectra
P60712	179.67	DSYVGDEAQSKR	SILAC 1	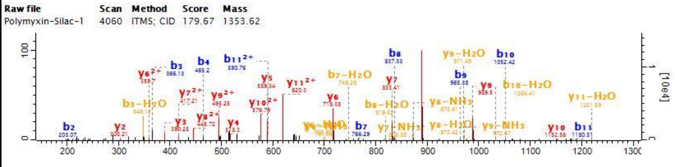
K4DKJ6	106.04	MLQQLQNMR	SILAC 1	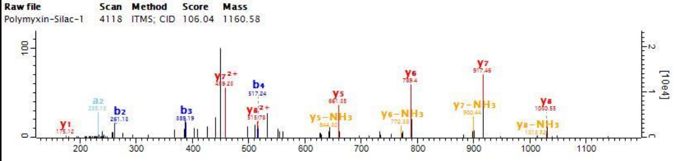
Q4E0K3	46.663	AMMVLLR	SILAC 2	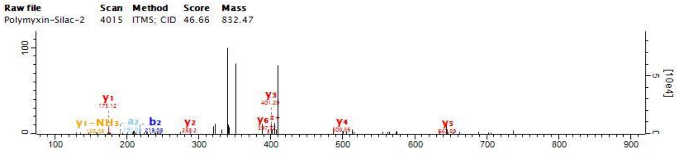
Q4E2S5	5.6511	HLGQVMEAYVSGEHSISSSNGSPLNLSK	SILAC 2	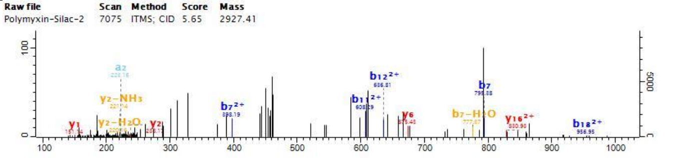
K4EBF6	1.855	QYYQLADWVVLVEVCTTQPPITETWVQSVDR	SILAC 2	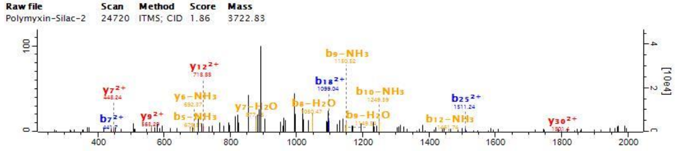
Q4CX61	15.667	YPCLPEEPAPGVAIVEVGLMEDPVFR	SILAC 2	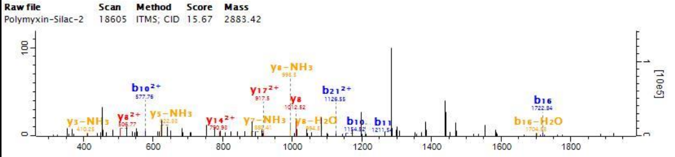

Table S3 List of peptides identified in polymyxin acylase experiments with their corresponding MaxQuant peptide scores.

Table S4 Enrichment of the *N*-myristoylome from parasites treated with DDD85646. Data is provided as an .xls file.

Table S5 Functional analysis of consistently enriched proteins. Data is provided as an .xls file.

UniProt Accession	NMT Eukaryota		Myristoylator
K4DJS2	RELIABLE	RELIABLE	High Confidence
K4DQN8	TWILIGHT ZONE	TWILIGHT ZONE	Not myristoylated
K4DT87	RELIABLE	RELIABLE	High Confidence
K4DTB6	NO	NO	High Confidence
K4DTI6	NO	NO	High Confidence
K4DTV5	TWILIGHT ZONE	TWILIGHT ZONE	High Confidence
K4DUN8	RELIABLE	RELIABLE	High Confidence
K4DV27	NO	NO	High Confidence
K4DVI8	RELIABLE	TWILIGHT ZONE	Medium Confidence
K4DWF7	RELIABLE	RELIABLE	High Confidence
K4DWR5	RELIABLE	TWILIGHT ZONE	High Confidence
K4DX27	NO	NO	Not myristoylated
K4DXD3	NO	NO	Not myristoylated
K4DXT8	TWILIGHT ZONE	NO	High Confidence
K4DZS1	RELIABLE	RELIABLE	Medium Confidence
K4E189	NO	NO	Not myristoylated
K4E1X7	RELIABLE	TWILIGHT ZONE	High Confidence
K4E3U8	RELIABLE	RELIABLE	High Confidence
K4E3X3	NO	NO	Not myristoylated
K4E583	NO	NO	High Confidence
K4E595	RELIABLE	RELIABLE	High Confidence
K4E5N2	RELIABLE	RELIABLE	High Confidence
K4E5P0	TWILIGHT ZONE	RELIABLE	High Confidence
K4E5Y1	RELIABLE	RELIABLE	High Confidence
K4E6H0	NO	NO	High Confidence
K4E6H6	TWILIGHT ZONE	NO	Medium Confidence
K4E818	RELIABLE	RELIABLE	High Confidence
K4E8V8	RELIABLE	RELIABLE	High Confidence
K4E943	RELIABLE	TWILIGHT ZONE	High Confidence
K4E955	RELIABLE	RELIABLE	High Confidence
K4EC97	RELIABLE	RELIABLE	Not myristoylated
K4EE92	RELIABLE	NO	Not myristoylated
K4EEE5	RELIABLE	RELIABLE	High Confidence
Q4CV42	RELIABLE	RELIABLE	High Confidence
Q4CW64	RELIABLE	RELIABLE	High Confidence
Q4CZT4	RELIABLE	RELIABLE	Low Confidence
Q4D0B9	NO	NO	Low Confidence
Q4D708	RELIABLE	RELIABLE	High Confidence
Q4D7Y8	NO	NO	High Confidence
Q4DDD2	RELIABLE	RELIABLE	High Confidence
Q4DLX6	TWILIGHT ZONE	NO	Not myristoylated
Q4DPA5	RELIABLE	RELIABLE	High Confidence
Q4DPJ1	NO	NO	High Confidence
Q4DRI6	TWILIGHT ZONE	TWILIGHT ZONE	High Confidence
Q4DVL2	TWILIGHT ZONE	TWILIGHT ZONE	Not myristoylated
Q4DXG4	TWILIGHT ZONE	TWILIGHT ZONE	Not myristoylated
Q4DZM9	TWILIGHT ZONE	TWILIGHT ZONE	Medium Confidence
Q4E2Z0	RELIABLE	NO	Low Confidence
Q4E4N2	NO	NO	Not myristoylated
K4DQW3	NO	NO	Not myristoylated
K4DYH4	No MG	No MG	No MG
K4E0J9	No MG	No MG	No MG
K4E0P3	No MG	No MG	No MG
K4E8Y0	No MG	No MG	No MG
Q4CWV8	No MG	No MG	No MG
Q4DEA8	No MG	No MG	No MG

Table S6 Prediction of N-myristoylation using Myristoylator and the NMT Myr predictor with the eukaryota (without fungi) or fungi-specific settings. Low and medium confidence predictions in Myristoylator were assigned the same significance as the twilight zone predictions from the NMT predictor.

Table S7. Identifying orthologs of *N*-myristoylated proteins from similar enrichments from *Plasmodium falciparum* and *Leishmania major*. Data is provided as an .xls file.
